# 

**Published:** 1905-08

**Authors:** 


**This Journal and Atlanta Semi-weekly Journal one year for $1.25 cash in advance. No checks. Strictly to new subscribers.**

